# False lumen thrombus following aortic dissection diagnosed as the source of repeat lower extremity emboli with angioscopy: a case report

**DOI:** 10.1186/s40792-022-01416-7

**Published:** 2022-04-13

**Authors:** Riha Shimizu, Makoto Sumi, Yuri Murakami, Takao Ohki

**Affiliations:** 1 Department of Vascular Surgery, Saitama Prefectural Cardiovascular and Respiratory Center, 1696, Itai, Kumagaya City, Saitama Prefecture 360-0197 Japan; 2 Division of Vascular Surgery, Department of Surgery, The Jikei University School of Medicine, 3-19-18, Nishishinbashi, Minato-ku, Tokyo, 105-8471 Japan

**Keywords:** Chronic aortic dissection, Acute lower extremity arterial occlusion, Non-obstructive angioscopy, Coil embolization

## Abstract

**Background:**

Acute arterial embolization caused by a free-floating thrombus of the false lumen after surgery for acute aortic dissection is a rare complication; hence, determining its cause may be difficult. We report a case in which angioscopy was valuable in diagnosing and treating the unstable thrombus within the false lumen.

**Case presentation:**

The patient was a 71-year-old woman who underwent hemiarch replacement for Stanford type A acute aortic dissection. Two months after the operation, left renal infarction occurred. Eighteen months after the operation, the patient visited the hospital for treatment of intermittent claudication of her left leg. Computed tomography (CT) showed occlusion below the left common femoral artery. Surgical thrombectomy was performed for acute lower extremity arterial occlusion. One month later, thrombectomy was performed again for the same phenomenon and again after 2 months. She had no history of cardiac arrhythmia. No obvious source for the repeat embolization could be found on echocardiography or enhanced CT. Angiography was performed to further identify the cause, revealing a new entry site at the distal anastomosis, which exhibited antegrade flow into the false lumen. Furthermore, selective false lumen angiography via the re-entry revealed a thrombus in the false lumen corresponding to the descending aorta. A non-obstructive angioscopy system in the false lumen revealed a free-floating thrombus. As the patient had undergone multiple surgeries over a short period and desired minimally invasive treatment, coil embolization of the new entry site as well as false lumen was performed. As a result, blood flow from the true to the false lumen resolved. More than 1 year has passed following coil embolization with no signs of embolism.

**Conclusions:**

We experienced a case of repeat embolism caused by unstable thrombus formation in the false lumen resulting from antegrade blood flow in the false lumen secondary to development of a new entry site. Angioscopy revealed that this antegrade flow caused formation of an unstable thrombus which caused recurrent acute lower extremity arterial occlusion. Therefore, angioscopy may be a useful option for the diagnosis of false lumen thrombosis.

## Background

Complications after acute aortic dissection surgery include enlargement and rupture of the false lumen and branch vessel ischemia [1, 2]. There have been few reports on thrombotic obstruction caused by a free-floating thrombus of the false lumen [3, 4], and determining its cause can be difficult. We used a non-obstructive angioscopy system to diagnose the presence of an unstable thrombus within the false lumen secondary to formation of a new entry site. We believe that this unstable thrombus that was otherwise difficult to diagnose lead to recurrent acute lower extremity arterial occlusions.

## Case presentation

A 71-year-old woman with a history of hypertension, subarachnoid hemorrhage, and hemiarch replacement for Stanford type A acute aortic dissection came to the hospital for intermittent claudication of her left leg. She had a history of left renal infraction 2 months after the operation.

**Fig. 1:**
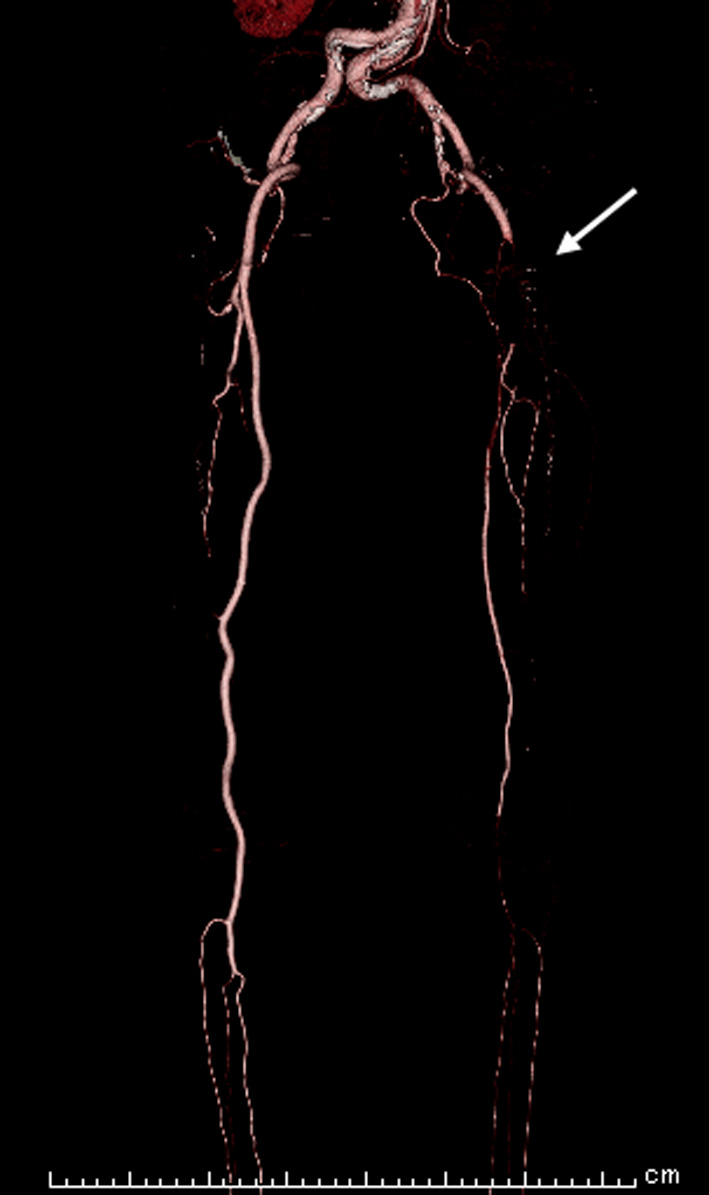
Preoperative computed tomography. Preoperative computed tomography shows thrombi in the left common femoral, superficial and deep femoral, and popliteal arteries (arrow)

**Fig. 2:**
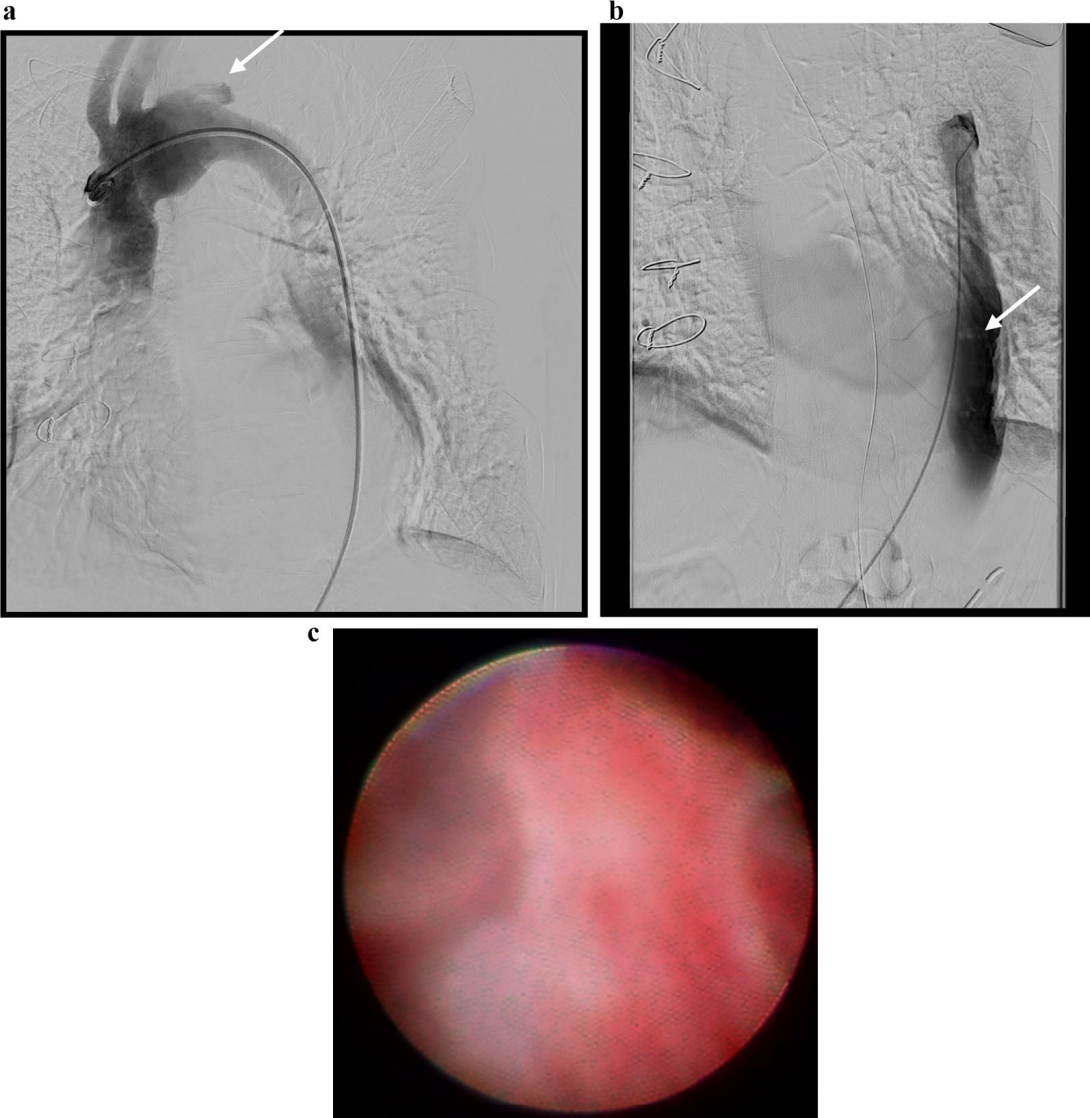
Preoperative angiography and intraoperative angioscopy. **a** Aortography shows blood flow into the false lumen, and shows a new entry site from the true to the false lumen near the distal side of the anastomosis (arrow). **b** Contrast enhancement of the false lumen shows stagnation of blood in the distal part, and indicates a thrombus in the false lumen (arrow). **c** Angioscopy of the false lumen shows a free-floating thrombus

The left femoral artery was pulseless, there were no signs of paresthesia or paralysis; however, she reported symptoms of pain and coldness in her left foot. Electrocardiogram showed sinus rhythm; echocardiography showed normal cardiac function, with no obvious intra-cardiac thrombus formation or enlargement of the left atrium. Computed tomography (CT) revealed occlusion from the left common femoral artery to the superficial and deep femoral, and popliteal arteries (Fig. 1). A diagnosis of acute arterial occlusion of the left lower extremity was made, and emergency thrombectomy was performed on the day of arrival at the hospital. Warfarin was administered postoperatively (target PT-INR 1.8–2.5). However, 1 month later, the pain in the left leg recurred. Because the same findings were observed, emergency thrombectomy was performed the same day. Warfarin and aspirin (100 mg/day) were administered. Finally, the left leg pain recurred 1 month later, and thrombectomy was repeated. CT scan revealed the entry site was the distal anastomosis, the re-entries were in the left common iliac artery, and some micro re-entries. Aortography revealed a new entry site near the distal anastomosis, and contrast imaging of the false lumen indicated the formation of a thrombus (Fig. 2a, b). Using non-obstructive angioscopy, a VISIBLE Fiber (Fiber Tech Co., Ltd., Tokyo, Japan) and a Fiber Imaging System FT-203F (Fiber Tech Co., Ltd., Tokyo, Japan) was inserted into the false lumen via the distal re-entry site. Angioscopy revealed the presence of a free-floating thrombus inside the false lumen (Fig. 2c); a portion of the thrombus was collected by suctioning with a TEMPO™ catheter (Cordis, Cardinal Health, Ohio, USA). Blood flow from the tear in the distal part of the anastomosis was determined to have caused the thrombus; hence, the treatment plan included occlusion of the entry site. We performed entry site closure from the false lumen side.

**Fig. 3:**
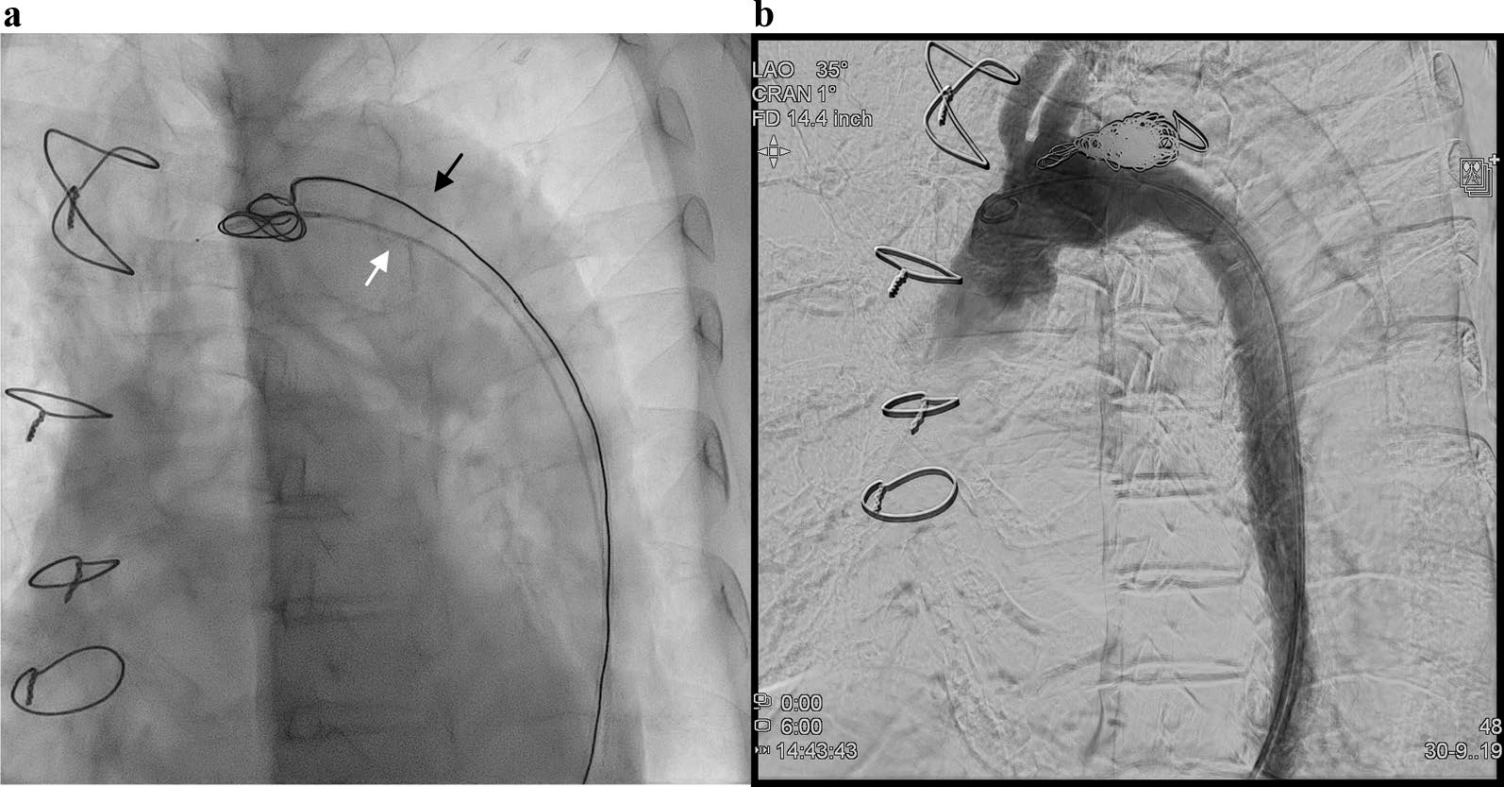
Intraoperative angiography. **a** A catheter is inserted, and an Interlock-35 is placed as an anchor (black arrow). Next, a microcatheter is inserted via another catheter to perform coil embolization (white arrow). **b** The final aortography shows lack of blood flow in the false lumen. Final contrast enhancement shows no blood flow from the true to the false lumen

**Fig. 4:**
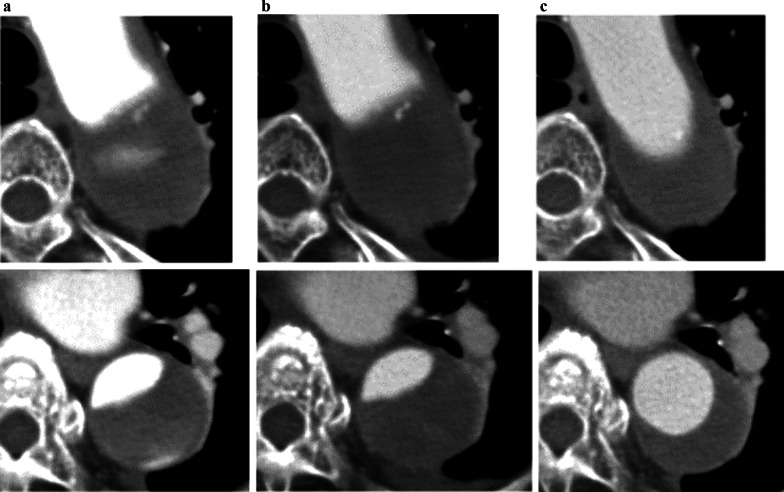
Preoperative and postoperative computed tomography. **a** Blood flow is observed in the false lumen in preoperative CT. **b** In an early postoperative CT, blood flow has disappeared from the false lumen. **c** In a CT 1-year postoperatively, the diameter of the vessel has reduced, and the true lumen has increased in size

Under local anesthesia, a 5-Fr sheath and a 5-Fr Destination™ guiding sheath (Terumo, Tokyo, Japan) were inserted via the left common femoral artery. A TEMPO™ catheter and a Renegade™ microcatheter (Boston Scientific, Marlborough, MA, USA) were placed in the false lumen via the 5-Fr sheath; and another TEMPO™ catheter was inserted into the false lumen via the 5-Fr Destination™ guiding sheath; an Interlock-35 (Boston Scientific, Marlborough, MA, USA) was placed as an anchor; thus, the embolization was performed using the Interlock-35 via the microcatheter (Fig. 3a). As a result, the blood flow from the true to the false lumen resolved (Fig. 3b). The postoperative course was uneventful, and the patient was discharged after 8 days. Follow-up CT showed the progression of thrombus formation in the false lumen, and aortic remodeling including shrinkage of the false lumen (Fig. 4a–c); pathological findings of the thrombus from the first thrombectomy resembled those of the thrombus suctioned from the false lumen catheter. Warfarin and aspirin were stopped after coil embolization. There were no signs of embolism and coil migration at the 1-year follow-up since the obliteration of the entry site.

## Discussion

We report a case of recurrent embolism, after the hemiarch replacement for acute Stanford type A aortic dissection. However, this was not clear on diagnostic imaging, including enhanced CT, and the embolic source could only be confirmed by angioscopy of the false lumen. To the best of our knowledge, this is the first report of such a case.

Eighty percent of re-operations after acute aortic dissection surgery are due to rupture, enlargement of aortic aneurysm, or re-dissection [1, 5]. Reports of embolism caused by a free-floating thrombus are rare [3, 4]. While believed to involve thrombi that formed in the false lumen owing to the antegrade blood flow of the residual dissection, some reported cases were diagnosed when other differential diagnoses were ruled out [4, 6]. In the present case, we searched for the source of the embolism; atrial fibrillation, cardiac tumor, or other factors were ruled out. CT and angiography showed antegrade blood flow from the distal part of the anastomosis to the false lumen. The large re-entry site at the left common iliac artery, and the three incidents of left lower extremity occlusion indicated false lumen thrombosis [1, 2]. Angiography showed blood flow from entry site into the false lumen stagnated in false lumen, therefore, we thought that blood flow from entry site might indicate a thrombus in the false lumen. However, in order to gain definitive diagnosis, we decided to perform angioscopy because a diagnosis could not be made using diagnostic imaging such as CT and magnetic resonance imaging (MRI).

Angioscopy has been covered by health insurance since 2016 in Japan. It is typically used to diagnose arterial diseases, along with CT and MRI; though angioscopy may lead to plaque and vascular damage. However, angioscopy can be used for invasive, direct observations of vessels ≥ 2 mm, including the thoracoabdominal aorta, and the subclavian, renal, iliac, femoral, and pulmonary arteries. These observations are performed to evaluate local arteriosclerosis; to assess the effects of treatment on an artery; for postoperative evaluations and follow-up; to estimate the timing of thromboembolism from pulmonary embolism [7]; and to check for tears or ulcer-like projection lesions in cases of aortic dissection [8, 9]. In the present case, angioscopy inserted into the false lumen of the aortic dissection tear was used to search for the embolic source. Complications of angioscopy include cerebral infarction, although rare [7], and the procedure is considered relatively safe. Intravascular ultrasound (IVUS) is also covered by medical insurance. The patient in this case had requested that the procedure be performed under local anesthesia. We had selected angioscopy for two reasons: first, the narrow diameter of the angioscopy sheath (5-Fr); second, we had thought that angioscopy would be more useful in investigating the cause of the thrombus, since surface texture can be ascertained by using it.

In addition, recurrent embolisms are sometimes encountered in cases of abdominal aortic aneurysms; however, these are usually discovered on CT, the typical finding of which includes the disappearance of a thrombus in an aneurysm. Therefore, in cases of chronic dissection, such as in the present case, it may be difficult to make a definitive diagnosis with CT. Therefore, to confirm whether the false lumen thrombus was consistent with embolic material, a catheter was inserted to obtain a sample of the thrombus. The pathological findings of the thrombus collected from the false lumen resembled the characteristics of free-floating thrombus. Thus, we diagnosed it as a thrombus that had become detached from the false lumen. Inserting the catheter directly into the false lumen allowed us to collect the tissue by suctioning the thrombus.

Anticoagulants reportedly improve embolisms caused by free-floating thrombi in chronic aortic dissection [4, 10]; however, surgery was undertaken in the present case because the patient experienced repeated embolic events, even though she was on oral anticoagulant and antiplatelet therapy. The purpose of the treatment was the prevention of the embolism, and it was necessary to close the entry site. In cases of an enlarged false lumen, re-operation with aortic arch replacement or debranching using thoracic endovascular aortic repair (TEVAR) can be considered; additionally, descending aortic replacement can also be considered to remove the source of the emboli. However, aortic arch and descending aortic replacement are both highly invasive procedures, and there are significant risks associated with re-operations. Stent-graft therapy with closure of the entry site is a minimally invasive and effective treatment. However, closure of the entry site alone does not shrink an enlarged false lumen; instead, it may continue to expand with an increased risk of rupture [1, 11]. A false lumen can be occluded using coil embolism, candy plug, vascular plug, or knickerbocker techniques [1, 12]. A therapeutic effect can be expected if complete exclusion of an enlarged false lumen is achieved [13]. In the present case, it had been more than a year since the onset, and while the false lumen was partially thrombosed, the diameter of the aneurysm was only 35 mm. When embolizing via the true lumen, a 2-debranch TEVAR is necessary. Therefore, preemptive TEVAR was not indicated due to the absence of aneurysm enlargement [14], and TEVAR was judged to be over treatment in the present case.

The suppression of blood flow from the true to the false lumen may significantly reduce blood flow in the false lumen, thereby preventing the formation of an unstable thrombus and subsequent embolic events. Therefore, we believed that coil embolization of the false lumen was the best available treatment option. We had inserted two catheters, and used one of the coils as an anchor coil, without releasing it. We had performed coil embolization via the second catheter by maneuvering to wrap it around the anchor coil, to avoid migration. In addition, the patient had already undergone several surgeries in recent months and desired minimally invasive treatment under local anesthesia; hence, coil embolization of the false lumen was performed under local anesthesia. As a result, the false lumen blood flow and recurrent embolic events resolved. At the 1-year follow-up, the aneurysm was shrinking, and further embolisms have been prevented.

## Conclusions

We experienced a case of embolism due to false lumen partial thrombosis caused by antegrade blood flow in the false lumen from a new entry site. This flow and the subsequent false lumen thrombus resulted in recurrent acute lower extremity arterial occlusion with no apparent embolic source on conventional diagnostic imaging. Diagnosing of free-floating thrombus can be difficult; hence, angioscopy may be useful for making the diagnosis. In addition, coil embolization of the false lumen can suppress blood flow to the false lumen, and it is a minimally invasive, safe, and effective treatment procedure.

## Data Availability

Not applicable.

## Abbreviations

CT: Computed tomography
MRI: Magnetic resonance imaging
TEVAR: Thoracic endovascular aortic repair
